# Genome of the bee *Holcopasites calliopsidis—*a species showing the common apid trait of brood parasitism

**DOI:** 10.1093/g3journal/jkac160

**Published:** 2022-06-28

**Authors:** Trevor J L Sless, Jeremy B Searle, Bryan N Danforth

**Affiliations:** Department of Ecology and Evolutionary Biology, Cornell University, Ithaca, NY 14853, USA; Department of Ecology and Evolutionary Biology, Cornell University, Ithaca, NY 14853, USA; Department of Entomology, Cornell University, Ithaca, NY 14853, USA

**Keywords:** *Holcopasites calliopsidis*, brood parasite, bees, Hymenoptera, genome size, gene family evolution

## Abstract

Brood parasites represent a substantial but often poorly studied fraction of the wider diversity of bees. Brood parasitic bees complete their life cycles by infiltrating the nests of solitary host bees thereby enabling their offspring to exploit the food provisions intended for the host’s offspring. Here, we present the draft assembly of the bee *Holcopasites calliopsidis*, the first brood parasitic species to be the subject of detailed genomic analysis. Consistent with previous findings on the genomic signatures of parasitism more broadly, we find that *H. calliopsidis* has the smallest genome currently known among bees (179 Mb). This small genome does not appear to be the result of purging of repetitive DNA, with some indications of novel repetitive elements which may show signs of recent expansion. Nor does *H. calliopsidis* demonstrate any apparent net loss of genic content in comparison with nonparasitic species, though many individual gene families do show significant contractions. Although the basis of the small genome size of this species remains unclear, the identification of over 12,000 putative genes—with functional annotation for nearly 10,000 of these—is an important step in investigating the genomic basis of brood parasitism and provides a valuable dataset to be compared against new genomes that remain to be sequenced.

## Introduction

Though bees are particularly well-known for eusociality, this way of life is in fact only seen in about 10% of bee species (Danforth *et al.* 2019). Nonsocial bees demonstrate a wide range of behavioral strategies including specialized plant associations, diverse nesting strategies, and the parasitic exploitation of other bees. With over 2,700 species, bees include a higher proportion of brood parasites than any animal taxon of comparable size (Sless *et al.*, in review). Yet despite this prevalence, brood parasitic species have received relatively little attention, including in the field of genomics. While some 70 bee species now have publicly available genomes through GenBank, nearly three-quarters of these represent social species (Sayers *et al.* 2020). Currently, 1 brood parasite (*Nomada fabriciana*) has been sequenced (Wellcome Sanger Institute 2021), however, this genome is unannotated and has not been the subject of further analysis.

Broad shifts in genomic organization and architecture have been discovered that can be associated with the evolution of sociality (Kapheim *et al.* 2015; Shell *et al.* 2021) and social parasitism (Schrader *et al.* 2021) in other groups of hymenopterans, and the evolution of brood parasitism similarly involves phenotypic and behavioral shifts which must have a genomic basis. Some general patterns in genomic evolution have been identified among parasitic animals more generally (reviewed by Poulin and Randhawa 2015). One of the clearest of these is a trend toward reduced genome size in parasitic species, which has also been noted for other parasitic hymenopterans (Ardila-Garcia *et al.* 2010; Sundberg and Pulkkinen 2015). The typical explanation for this pattern involves relaxed selection on parts of the genome necessary for survival in a free-living organism, but which may be effectively “off-loaded” by a parasite to its host. While this may involve the loss of metabolic or developmental genes in endoparasites that spend their entire lives within a host, parasitic Hymenoptera (including brood parasites) retain a free-living adult stage without losses in basic functionality. Despite this differing dynamic, brood parasites may be thought of as off-loading *behavioral* responsibilities rather than metabolic attributes (in the form of parental care through nest building and food provisioning) to the host organisms on which they rely for survival. The question then follows: do brood parasitic bees show similar patterns of broad-scale genomic evolution to other parasites?

The genus *Holcopasites* is a member of the oldest and most diverse group of brood parasitic bees, the subfamily Nomadinae (Apidae) and the only Nearctic representative of the tribe Ammobatoidini. The predominant species in northeastern North America, *Holcopasites* *calliopsidis*, is a specialist parasite of the solitary mining bee *Calliopsis andreniformis*. Though the study of this single species presents a limited opportunity for inferences about the evolutionary signatures of brood parasitism on insect genomes more generally, it represents an important starting point that can serve as the basis for further comparative study as more genomes become available. Therefore, we here provide broad-scale characterization of the genome of *H. calliopsidis*.

## Methods

### Specimen collection and DNA extraction

Individuals of *H.* *calliopsidis* were obtained in June of 2020 from the Lime Hollow Nature Center in Cortland, New York, USA (42.57°N, 76.25°W). Specimens were collected by hand-netting, flash-frozen in liquid nitrogen, and sexed with the assistance of a dissecting microscope before storing at −80°C. Only male specimens were used for subsequent steps, due to the benefit of their haploid genomes. High-molecular weight DNA extractions were conducted using a Qiagen 20/G genomic tip kit (catalog #10223) and associated buffers (catalog #19060). The protocol used followed that recommended by Qiagen’s Genomic DNA Handbook (accessed December 4th, 2020 from https://www.qiagen.com/us/resources/resourcedetail?id=d2b85b26-16dd-4259-a3a7-a08cbd2a08a3&lang=en). Briefly, whole specimens were mechanically homogenized using a sterile pestle before being treated with RNase A (NEB catalog #T3018L) and proteinase K for 2 h. The resulting lysate was added to the equilibrated genomic tip columns, washed 3 times, and eluted. The eluted genomic DNA was then washed one more time with 70% ethanol before final resuspension in TE buffer. Samples were then assayed for quality and DNA concentration using a Qubit 4 fluorimeter.

### Sequencing and assembly

Four samples were sent to the University of Maryland’s Institute for Genome Sciences for sequencing. These were processed using Pacific Biosciences’ low-input library preparation and analyzed for DNA quality and size distribution. The highest-quality sample was then sequenced with a 30-h run on a Sequel II 8M SMRT Cell in CCS/HiFi mode. Raw data, subreads, and circular consensus sequences were subsequently received from the sequencing facility. The *H. calliopsidis* genome was assembled from the CCS reads described above using SPAdes v3.14.0 (Bankevich *et al.* 2012) in “assembler only” mode. The resulting assembly was analyzed for completeness and quality using QUAST v5.0.2 (Gurevich *et al.* 2013) and BUSCO v5.0.0 (Seppey *et al.* 2019).

### Repetitive element identification

The genome was searched for repetitive content using 2 passes of the program RepeatMasker v4.1.0 (Smit *et al.* 2013–2015) in softmasking mode. An initial run was conducted using a canonical repeat library from RepBase, DFam 5.0 (Storer *et al.* 2021). The result was then passed into a second run of RepeatMasker with a custom, species-specific repeat library generated using RepeatModeler v2.0.1 (Flynn *et al.* 2020).

### Gene annotation

The repeat-masked genome was annotated using BRAKER v2.1.5 (Brůna *et al.* 2021) in “EP” mode, along with a database of orthologous arthropod protein sequences obtained from ensembl.org (Howe *et al.* 2021). Annotation files from different lines of evidence within the BRAKER2 pipeline (Augustus and GeneMark) were combined using EVidenceModeler v1.1.1 (Haas *et al.* 2008) to generate a final annotation file. Transcripts and protein sequences were then extracted from the genome with the software GffRead (Pertea and Pertea 2020) using this annotation file as a guide.

### Ortholog detection

Proteomes from 13 other hymenopteran species (*Habropoda laboriosa*, *Apis mellifera*, *Bombus impatiens*, *Ceratina calcarata*, *Osmia bicornis*, *Megachile rotundata*, *Megalopta genalis*, *Nomia melanderi*, *Camponotus floridanus*, *Polistes dominula*, *Chelonus insularis*, *Nasonia vitripennis*, and *Athalia rosae*), with *Drosophila melanogaster* as an outgroup, were obtained from GenBank for comparison with newly annotated protein sequences from the *H. calliopsidis* genome. The software OrthoFinder v2.5.4 (Emms and Kelly 2019) was used to identify orthogroups among these protein sequences, running with default settings and using DIAMOND (Buchfink *et al.* 2015) for sequence comparisons.

### Gene family evolution

Expansion and contraction of gene families was analyzed with CAFE v4.2.1 (Han *et al.* 2013) using the orthogroups detected by OrthoFinder as the input dataset. The initial species tree produced by OrthoFinder did not produce the correct topology in comparison with established phylogenies and cannot produce an ultrametric tree when provided with a preset topology. As a result, the time-calibrated tree required by CAFE was generated manually using divergence time estimates from a previous phylogenomic study (Peters *et al.* 2017).

### Functional analysis

Initial gene ontology annotations were conducted with both InterProScan v5.51-85.0 (Jones *et al.* 2014) and DIAMOND v2.0.9 (Buchfink *et al.* 2015) using the Uniref90 reference dataset. The resulting .xml files from both programs were passed on to Blast2GO v1.5.1 (Götz *et al.* 2008). Functional enrichment analyses were conducted in Cytoscape (Shannon *et al.* 2003) using the BiNGO application v3.0.4 (Maere *et al.* 2005) with a custom annotation file modified from the Blast2GO output.

## Results

### Genome assembly

**Fig. 1.**
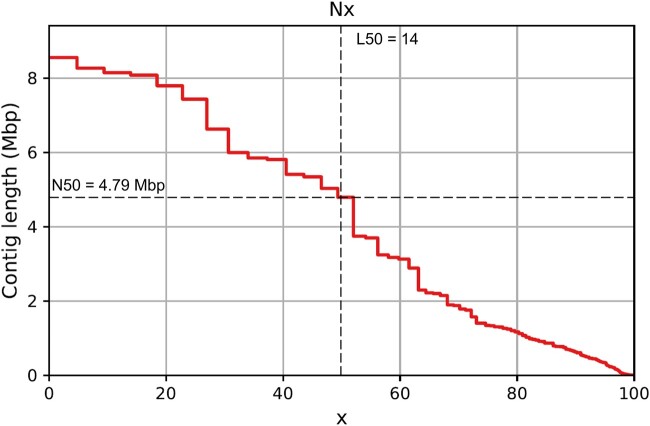
“Nx plot” generated by QUAST (Gurevich *et al.* 2013) showing contigs ranked by size vs cumulative assembly completeness. N50 and L50 values (minimum contig size and minimum number of contigs containing 50% of the assembly, respectively) for the *H. calliopsidis* genome are indicated by horizontal and vertical dashed lines.

The final genome assembly after running SPAdes on the circular consensus sequencing data included 5,627 contigs, with 491 > 1 kb in length (Fig. 1). This resulted in a total assembly size of just over 179 Mb, corresponding to an expected *C*-value of 0.183 pg. Though the estimated coverage of 47× was lower than some other recent assemblies, the N50 value of nearly 4.8 Mb compares favorably (Table 1). The L50 value of 14 indicates that the largest several contigs likely represent entire chromosomes or major sections thereof, however the exact chromosome count and structure cannot be accurately determined from the assembly. The considerable number of extremely small scaffolds can be effectively ignored, as the smallest 5,188 of these (<1 kb) contain just 0.56% of the overall assembly despite making up 92.2% of all scaffolds. This genome assembly appears to be largely complete, with 97% of the 5,991 highly conserved single-copy orthologs in BUSCO’s Hymenoptera dataset identified (the remainder comprising 0.3% duplicated orthologs, 0.6% fragmented, and 2.1% not detected). It is also noteworthy that the genome of *H. calliopsidis* has one of the highest percentages of GC content of any known bee at approximately 42.5%.

**Table 1.** Comparison of assembly statistics for *H. calliopsidis* and recent assemblies from a selection of nonparasitic species spanning the diversity of bees (in approximate order of increasing phylogenetic distance from *H. calliopsidis*).

Species	Family: subfamily	Assembly size (Mb)	No. of scaffolds	N50 (bp)	Coverage (×)	Source
*Holcopasites calliopsidis*	Apidae: Nomadinae	179	5,627 (491 > 1 kb)	4,790,652	∼47	This study
*Habropoda laboriosa*	Apidae: Anthophorinae	297	27,566	1,784,116	113	Kapheim *et al.* (2015)
*Apis mellifera*	Apidae: Apinae	225	177	13,619,445	192	Wallberg *et al.* (2019)
*Bombus impatiens*	Apidae: Apinae	248	5,559	1,399,493	108	Sadd *et al.* (2015)
*Megachile rotundata*	Megachilidae: Megachilinae	273	6,266	1,699,680	272	Kapheim *et al.* (2015)
*Nomia melanderi*	Halictidae: Nomiinae	299.6	3,194 > 1 kb	2,054,768	75	Kapheim *et al*. (2019)
*Colletes gigas*	Colletidae: Colletinae	273	326	8,109,000	147.5	Zhou *et al.* (2020)

### Genome size

**Fig. 2.**
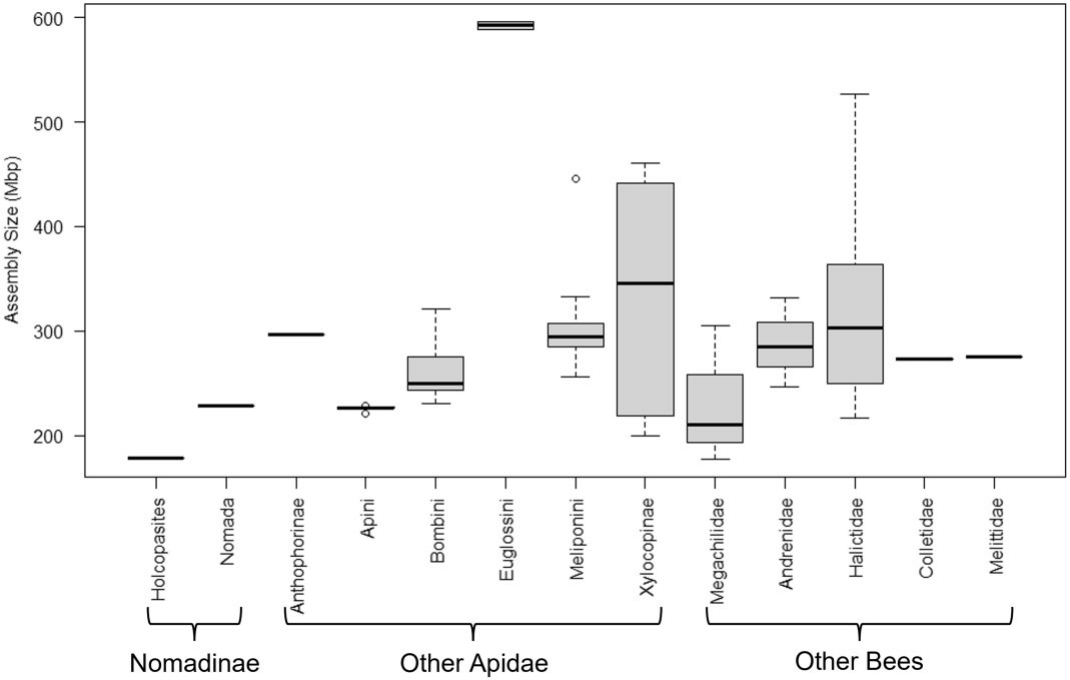
Size comparison in megabases of all bee species with genome assemblies available on GenBank (*n* = 70), arranged by approximate phylogenetic distance from *H. calliopsidis*.

Based on a comparison of other assemblies published in GenBank, the 179 Mb *H. calliopsidis* genome assembly is the smallest of any bee species (Fig. 2), and among other Hymenopterans is exceeded in this respect only by some parasitoid wasps of the family Braconidae. The high proportion (97%) of orthologs detected by the BUSCO analysis above indicates that this result is not simply an artifact due to missing DNA, coverage problems, or a poor assembly. The true genome size should therefore be very close to the size of this assembly, and possibly even smaller if some trailing contigs represent microbial sequences rather than *H. calliopsidis* DNA.

### Repetitive content

The initial run of RepeatMasker using the Dfam canonical repeat library identified a relatively small proportion of repetitive sequences in the *H. calliopsidis* genome. Less than 1% of the assembly is composed of known noninterspersed elements including satellite sequences and short simple repeats (SSRs), in contrast to ∼4% in the genome of the Western honey bee, *A.* *mellifera*. Similarly, *H. calliopsidis* has proportionately about half as many canonical retroelements than *A. mellifera* (2.12% vs 4.18%), though slightly more DNA transposons in direct comparison (1.79% vs 0.57%). However, the second run using a species-specific library produced by RepeatModeler identified a sizeable proportion of the *H. calliopsidis* genome as “unclassified” repeats not yet represented in any database of repetitive sequences, comprising a total of nearly 14% of the assembly. These unclassified repetitive elements specifically included a total of 698 distinct families with an average size of 200.9 bp and average of 154.6 copies masked throughout the genome.

### Gene annotation

The BRAKER2 pipeline identified 13,028 putative protein sequences derived from 12,364 predicted genes throughout the *H. calliopsidis* genome. Overall, this is similar to the number of genes reported from most other bee species. Though exact identity of these genes is often not possible to ascertain, the overall number and density indicates no net loss of genes in the *H. calliopsidis* genome in contrast to nonparasitic bees. Additionally, the average number and length of introns is similar between *H. calliopsidis* and other species. For example, *H. calliopsidis* averages 5.34 introns of 524.3 bp each per transcript, while the comparable figures for the genome of *Colletes gigas* average 4.99 introns of 665 bp each (Zhou *et al.* 2020), resulting in comparable proportions of intronic content (62% and 67% of overall genic content, respectively).

### Orthology detection

**Fig. 3.**
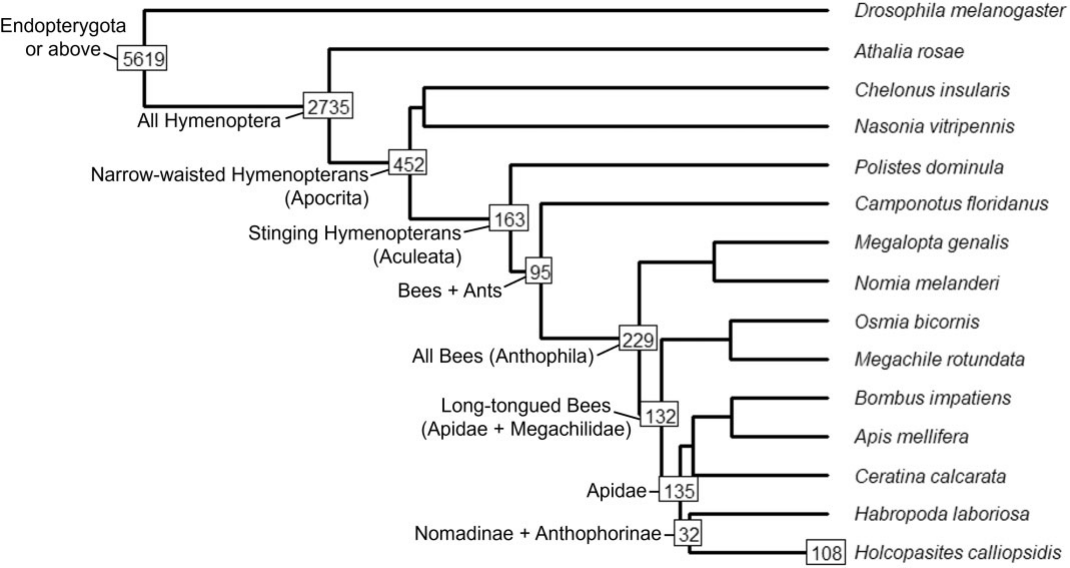
Phylogeny of *H. calliopsidis* and 14 additional species used in orthology analysis. Boxes at nodes indicate the number of orthogroups containing at least 1 *H. calliopsidis* gene (9,700 in total) which were phylogenetically conserved at the level of the indicated clade. The box at the tip of the *H. calliopsidis* branch indicates orthogroups not found in any other species. Branch lengths are to scale, with approximate divergence times taken from Peters *et al.* (2017).

OrthoFinder identified a total of 21,872 orthogroups across the proteomes of the included species (14 hymenopterans + *D.* *melanogaster*), which together accounted for 97.5% of all genes. The mean number of genes per orthogroup was 15.4 (median = 7), and a total of 4,298 orthogroups contained genes from every included species (of which 101 were single-copy orthogroups). A total of 10,913 putative genes from the *H. calliopsidis* genome were placed into exactly 9,700 orthogroups which were conserved at a range of phylogenetic scales (Fig. 3). Although this is similar to the average number of orthogroups for all species (mean = 9,910.47), *H. calliopsidis* had a noticeably higher fraction of genes which could not be assigned to any orthogroup (16.2%) than other species.

A total of 108 orthogroups containing 393 genes were identified that appear to be unique to the *H. calliopsidis* genome—henceforth referred to as “*H. calliopsidis*-specific” genes. An important caveat of this terminology is that, due to the taxonomic bias of proteomes available for comparison, it is not possible to determine whether these genes are truly unique to *H. calliopsidis*, or more broadly conserved across the entire genus *Holcopasites*, tribe Ammobatoidini, subfamily Nomadinae, or an intermediate clade between these. However, these numbers are somewhat smaller than the overall mean of 390.6 species-specific orthogroups (and 1,570 species-specific genes) among all 14 hymenopteran genomes, despite the longer branch length of *Holcopasites* in contrast to several other included bees. Outside of the Nomadinae, 32 orthogroups were shared with their closest sister group (digger bees in subfamily Anthophorinae), 135 conserved across the family Apidae, 229 conserved across all bees, and 2,735 shared across all Hymenoptera. However, the majority of orthogroups containing *H. calliopsidis* genes (5,619) were identified as conserved at the level of holometabolous insects (represented by *D. melanogaster*) or higher.

### Gene family evolution

**Fig. 4.**
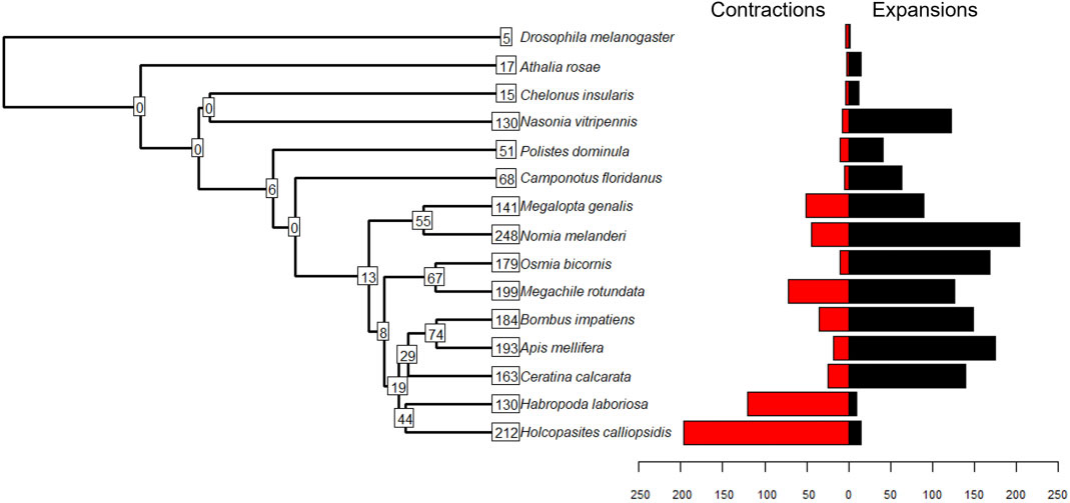
Phylogeny of *H. calliopsidis* and 14 additional species used in analysis of gene family evolution. Numbers at nodes and tips indicate how many rapidly evolving gene families were detected by CAFE for each species/lineage, respectively. Bars further separate rapidly evolving gene families for each tip into contractions or expansions in gene family size.

The orthogroups described above were further analyzed with CAFE to identify nodes in the phylogeny of the 15 included species with significant gene family expansions or contractions (Fig. 4). A single birth-death rate parameter (*λ* = 0.00259067) was estimated for the entire tree. Significantly elevated rates of gene family evolution (family-wide *P*-value < 0.01) were detected in 831 (3.80%) of orthogroups, with 212 rapidly evolving gene families identified in *H. calliopsidis* specifically (15 expansions, 197 contractions). In comparison with the other included species, this represents the second-highest number of rapidly evolving gene families (after *Nomia melanderi* with 248), but by far the most gene family contractions. Interestingly, when ranked by statistical significance, 9 of the top 10 gene families which have undergone the most rapid evolution in *H. calliopsidis* are expansions rather than contractions. Of these, several families include genes with blast2GO annotations suggesting involvement with transposable or retroviral elements, which may indicate a recent or ongoing spread of such features in the genome.

### Functional analysis

In total, InterProScan and blast2GO assigned over 60,000 GO terms to 9,943 genes annotated from the *H. calliopsis* genome, leaving about one-quarter of predicted genes without functional annotation. Analysis of the distribution of level 3 GO terms indicates few surprising features; various metabolic processes account for the most commonly assigned biological processes, and the most common molecular functions included enzymatic activity and binding of proteins and other compounds.

**Fig. 5.**
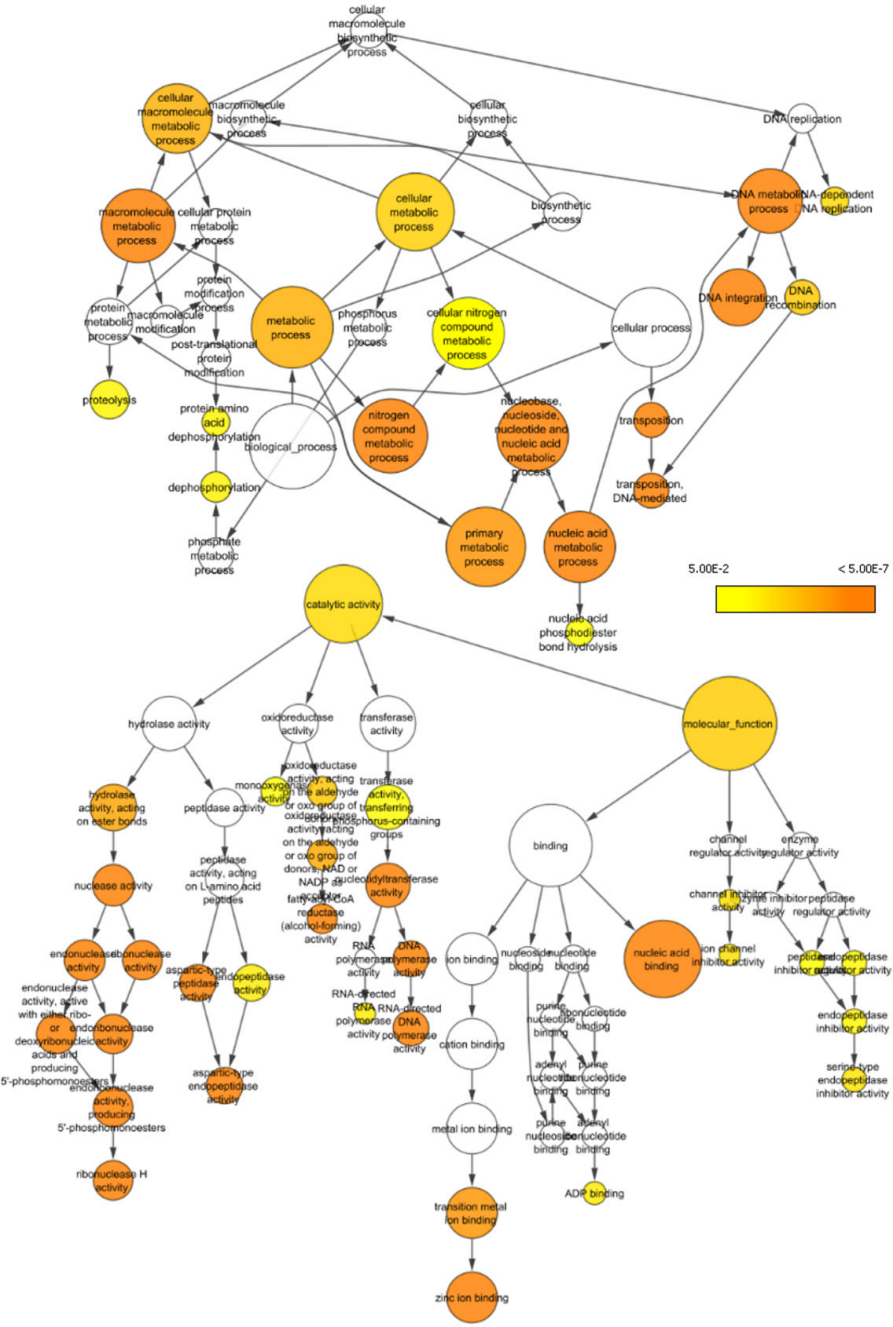
Selected sections of gene ontology network showing terms with significant enrichment among the set of 393 *H. calliopsidis*-specific genes compared to all annotated genes. Color gradient indicates degree of statistical significance from *P* = 0.05 (yellow) to *P* = 5 × 10^−7^ (dark orange).

A BiNGO analysis of the 393 *Holcopasites*-specific genes identified through orthology detection compared to the genome as a whole identified several GO terms that appear enriched for these loci (Fig. 5). Several of these (e.g. transposition, DNA integration, and various classes of endonuclease activity) may in fact be signatures of the large fraction of the genome containing presumed *Holcopasites*-specific repetitive elements as identified above.

A second BiNGO analysis compared the entire proteomes of our *H. calliopsidis* assembly with those of *Ha.* *laboriosa* (the phylogenetically closest well-annotated genome to *H. calliopsidis*) as well as *A.* *mellifera* to identify the GO terms which appear enriched in *H. calliopsidis* in contrast to its nonparasitic relatives or vice versa. Overall, this analysis was inconclusive. Many GO terms were identified as being significantly over- or underrepresented in *H. calliopsidis* in direct comparison to the other 2 species, yet in some cases these findings may be artifactual. Differences in the depth of gene ontology annotations achieved for the 3 genomes can result in artificial overrepresentation of GO terms which may not appear in all species, but which are simply child nodes of other well-represented terms. For example, GO terms for synthesis of all essential amino acids were identified as overrepresented in *H. calliopsidis* over *Ha. laboriosa*/*A. mellifera*, though it is difficult to explain this biologically. Some terms (e.g. neurogenesis/neuron development, oogenesis) were underrepresented in *H. calliopsidis* in ways which appear superficially consistent with a priori expectations about adaptations to the brood parasitic life history strategy. However, this must be considered with the caveat that enrichment of certain ontology terms, which may represent expansion of genes involved in a given biological process, is still separated from the actual control of such processes by several layers of regulation (e.g. transcriptional regulation, splicing, post-translational modifications).

## Discussion

The most striking feature of our broad-scale characterization of the *H. calliopsidis* genome is its small genome size, and consequently we focus the discussion on this attribute. However, as the first brood parasitic bee for which a well-characterized genome has been produced, there is much which remains to be learned. Brood parasitism has evolved at least 20 times independently in bees, with *H. calliopsidis* representing the oldest and most diverse such clade in the subfamily Nomadinae (Sless *et al.* in review). In this sense, our study has opened a new avenue of genomic research in relation to a well-known and fascinating behavioral strategy. This work is also complementary to nascent genomic studies on brood parasitic birds (such as cuckoos and cowbirds), which share a common life history despite their great phylogenetic distance from bees, and similarly include recently sequenced genomes available through GenBank but awaiting in-depth analysis (Wolf *et al.* unpublished data; Wuitchik *et al.* unpublished data; Zhang unpublished data).

### Genome size reduction

While most bee genomes fall into a fairly consistent range of ∼250–350 Mb, with a mean of 287.05 Mb for all 70 species with genomes available on GenBank, our assembly for *H. calliopsidis* is noticeably smaller at just 179 Mb. Methodological explanations for this finding seem unlikely, but corroboration of this estimate with cytological techniques could serve as an additional line of evidence. Assuming that the ancestral genome size for Apidae (or indeed for all bees) falls within the typical 250–350 Mb range, this represents a reduction of ∼25–45% in *H. calliopsidis*. The only other member of Nomadinae with a known genome size, *N.* *fabriciana*, falls on the lower end of the “typical” bee range at 233 Mb, indicating that at least some of this genomic contraction may be a recent change in the ancestors of *H. calliopsidis*, rather than a feature of all brood parasitic bees in the subfamily Nomadinae. Significant changes in genome size, especially when they occur over brief time periods, have often been attributed to the expansion or purging of repetitive elements (e.g. Hancock *et al.* 2021). Indeed, some bee lineages such as the orchid bees (Euglossini) appear to have undergone massive genomic expansions as a result of increased repetitive content (Brand *et al.* 2017). However, the reverse situation in which repeat sequences are purged from the genome does not appear to be the case in *H. calliopsidis*, which shows comparable or even higher levels of repetitive content to other species as a fraction of the genome. The large fraction of “unclassified” repetitive DNA which could not be matched to existing databases is likely due to the fact that this de novo genome assembly has no close relatives which have been previously analyzed for repetitive content. A similar phenomenon has been reported for at least one other phylogenetically distinct genome, that of *Colletes gigas*, which also consisted of >10% “unclassified” repetitive elements (Zhou *et al.* 2020).

Similarly, the reduced genome size in *H. calliopsidis* does not appear to be a result of net loss in genic content. Though many genes found in other species did not have orthologues annotated in *H. calliopsidis*, the overall number of putative genes identified was similar to other known bees at approximately 12,000. The average number and length of introns is also similar between *H. calliopsidis* and *A. mellifera*, which would seem to rule out any substantial purging of noncoding sequences within genes. However, this certainly does not mean that genic content is static or identical to that of other species. Many gene families are identified as having experienced rapid evolution in *H. calliopsidis*, though with the caveat that differing methodologies for gene annotation used across the compared species may artificially influence this analysis. It may be the case that *H. calliopsidis* does in fact exhibit the loss of a large number of genes associated with nonparasitic life histories, but that these losses are compensated to some extent by a number of rapidly evolving gene families. This finding may in fact be related to the large amount of “unclassified” repetitive elements discussed above, since transposable elements containing functional genes may be included among the most rapidly expanding gene families. It remains unclear exactly which types of material account for the reduction in *H. calliopsidis*’ genome size compared to the ancestral bee genome.

### Other features

Our genome assembly for *H. calliopsidis* is noticeably more GC rich (42.5%) than most other bee genomes. Of those available on GenBank, the mean GC content is 37.6%, and only 2 species (*Andrena dorsata* and *haemorrhoa*) surpass *H. calliopsidis* in this regard (Sayers *et al.* 2020). It is unclear what relation if any this may have to the evolution of brood parasitism, however. Proportion of GC bases is not generally correlated with genome size in animals (Li and Du 2014), though a positive correlation between GC content and recombination rates has been noted in honey bees (Beye *et al.* 2006).

The orthology detection analysis with OrthoFinder identified fewer unique orthogroups containing only *H. calliopsidis* genes than for any other included species besides *Ha.* *laboriosa*. This may initially seem to suggest that *H. calliopsidis* has experienced less evolution of novel genes than most other Hymenoptera. However, over 2,000 putative *H. calliopsidis* genes (a higher fraction than any other species) were not able to be assigned to any orthogroup, and some of these are surely also unique (but single-copy) genes that arose sometime after the split between Nomadinae and its sister group. Unsurprisingly though, the lack of interspecific orthologs for these unassigned genes also meant that the vast majority of them received no functional annotations from blast2GO, and so their identity remains unclear.

### Future directions

The annotated draft genome of *H.* *calliopsidis* presented herein is a major first step toward understanding the genomic basis of brood parasitism in the Nomadinae and in bees more broadly. However, as already addressed throughout this study, the amount of information which can be gained from a single genome is inherently limited. From this dataset alone, it is possible to identify many interesting features of genomic content in *H. calliopsidis*, but not their age or phylogenetic distribution as orthologs. Some such features may in fact be ancient signatures of the initial transition to brood parasitism in the subfamily Nomadinae approximately 100 MYA, while others could be much more recent innovations related to *H. calliopsidis*’ status as a narrow host specialist.

Future investigation into the genomics of brood parasitism should focus on sampling other species representing a wider phylogenetic distribution of parasites, including other members of the Nomadinae as well as bees representing independent origins of parasitism. Comparative genomic investigation has already yielded interesting results in studying the genomic basis of social behavior in bees (e.g. Kapheim *et al.* 2015; Shell *et al.* 2021), and it is our hope that the *H. calliopsidis* genome will serve as a starting point for the parallel exploration of the equally fascinating life history innovation of brood parasitism.

## Data availability

Our *H. calliopsidis* genome assembly is available through GenBank (accession # JALHAQ000000000). Data files resulting from major analyses (repetitive elements, gene annotations, functional annotations, and orthology/gene family investigations) have been uploaded to the GSA Figshare portal in association with this manuscript. Supplementary data files are available through the GSA figshare portal: https://doi.org/10.25387/g3.19522369. Supplementary File 1 (“Assembly”) includes the main contig-level assembly for *H. calliopsidis* as well as the results of a QUAST quality assessment and BUSCO analysis of genome completeness. Supplementary File 2 (“Repetitive Elements”) includes the masked assemblies resulting from 2 runs of RepeatMasker and RepeatModeler as described in the Methods section. Supplementary File 3 (“Annotation”) contains output gene annotation files from BRAKER2, protein, and transcript sequences extracted with EvidenceModeler and GffRead, and functional annotation output from InterProScan and Blast2GO, respectively. Finally, Supplementary File 4 (“Orthology”) provides the results of an OrthoFinder run to identify orthogroups among *H. calliopsidis* and other species, as well as an analysis of gene family evolution with CAFE (including the dated phylogeny used as input for this software based on Peters *et al.* 2017).
